# Stoichiometric Correlation Analysis: Principles of Metabolic Functionality from Metabolomics Data

**DOI:** 10.3389/fpls.2017.02152

**Published:** 2017-12-18

**Authors:** Kevin Schwahn, Romina Beleggia, Nooshin Omranian, Zoran Nikoloski

**Affiliations:** ^1^Systems Biology and Mathematical Modeling Group, Max Planck Institute of Molecular Plant Physiology, Potsdam, Germany; ^2^Bioinformatics Group, Institute of Biochemistry and Biology, University of Potsdam, Potsdam, Germany; ^3^Consiglio per la Ricerca in Agricoltura e L'analisi Dell'economia Agraria, Centro di Ricerca per la Cerealicoltura e le Colture Industriali (CREA-CI), Foggia, Italy; ^4^Center of Plant Systems Biology and Biotechnology, Plovdiv, Bulgaria

**Keywords:** metabolism, systems biology, maximal correlation, correlation analysis, domestication

## Abstract

Recent advances in metabolomics technologies have resulted in high-quality (time-resolved) metabolic profiles with an increasing coverage of metabolic pathways. These data profiles represent read-outs from often non-linear dynamics of metabolic networks. Yet, metabolic profiles have largely been explored with regression-based approaches that only capture linear relationships, rendering it difficult to determine the extent to which the data reflect the underlying reaction rates and their couplings. Here we propose an approach termed Stoichiometric Correlation Analysis (SCA) based on correlation between positive linear combinations of log-transformed metabolic profiles. The log-transformation is due to the evidence that metabolic networks can be modeled by mass action law and kinetics derived from it. Unlike the existing approaches which establish a relation between pairs of metabolites, SCA facilitates the discovery of higher-order dependence between more than two metabolites. By using a paradigmatic model of the tricarboxylic acid cycle we show that the higher-order dependence reflects the coupling of concentration of reactant complexes, capturing the subtle difference between the employed enzyme kinetics. Using time-resolved metabolic profiles from *Arabidopsis thaliana* and *Escherichia coli*, we show that SCA can be used to quantify the difference in coupling of reactant complexes, and hence, reaction rates, underlying the stringent response in these model organisms. By using SCA with data from natural variation of wild and domesticated wheat and tomato accession, we demonstrate that the domestication is accompanied by loss of such couplings, in these species. Therefore, application of SCA to metabolomics data from natural variation in wild and domesticated populations provides a mechanistic way to understanding domestication and its relation to metabolic networks.

## Introduction

Metabolomics profiling technologies are increasingly used for phenotyping of biological systems to understand the contribution of metabolism to complex phenotypes, including growth and diseases (Sumner et al., 2003; Schauer and Fernie, 2006; Kaddurah-Daouk et al., 2008). They have been used to assess the relative and absolute levels of different metabolites after perturbation or over time (Fiehn et al., 2000). The resulting metabolic data profiles manifest the joint effect of the rates of multiple biochemical reactions interrelated in metabolic networks. Reaction rates are themselves subjected to different types of regulation, often carried out by altering the concentration of metabolites (Koshland, 1970).

Regulation of reaction rates is necessary to ensure that the activities attributed to different parts of the system are coordinated. The simplest way to capture the coordination of reaction rates is through their coupling, whereby the ratio of the reaction rates is maintained in a narrow range (Millard et al., 2017), resulting in high positive correlation values between the coupled reaction rates over different experiments (e.g., environments). The principle questions in analyzing the data from metabolomics technologies are then to determine the extent to which the metabolite levels reflect the coupling of the underlying biochemical reactions as well as any differences in these characteristics between experimental scenarios (e.g., comparison of genotypes or treatments).

Despite the apparent non-linearites due to the metabolic structure and regulation, metabolic data profiles are usually analyzed by regression-based approaches that can only capture linear relationships. Ever since the seminal work of Vance et al. (2002), which used partial correlations to analyze the dependence between metabolites and reconstruct the reactions in which they participate, the existing analyses of metabolic data profiles rely on applying various similarity measures to given metabolic profiles (Çakir et al., 2009; Krumsiek et al., 2016). Since correlation, like other similarity measures, results in bilateral relationships between metabolites, the resulting metabolite-metabolite relationships have been represented and analyzed in the framework of metabolic correlation network analysis (MCNA) (Toubiana et al., 2013). This has led to the usage of MCNA to compare data from different scenarios based on the concept of differential networks (Chen et al., 2009; Ideker and Krogan, 2012). However, the principle question about coupling of biochemical reactions reflected in the metabolic profiles remains unresolved.

Assuming random fluctuations around a given steady state, metabolic correlations have been related to the Jacobian of the system of ODEs that describe the change in metabolite concentrations (van Kampen, 2007). In a series of studies, this relation has been employed for reconstructing the Jacobian of simplified metabolic networks and for comparison of different treatments (Steuer et al., 2003; Sun et al., 2015; Nägele et al., 2016). While this approach places metabolic correlations on strong theoretical basis, it is not applicable for analysis of instationary data. In another network-driven approach (Hackett et al., 2016), metabolic profiles have been fitted to steady-state compatible fluxes [extracted under optimality assumption of the flux balance analysis (Orth et al., 2010)] with different functional form for the reaction rates*v*(*x, k*). This approach has allowed the elucidation of novel regulators of reaction rates.

Here we take a principally different approach motivated by biochemically reasonable assumptions which often hold in realistic biological scenarios. Since biological systems sense and respond to environmental perturbations, they achieve normal functionality in face of these perturbations. To this end, various feedbacks and mechanisms based on network structure have evolved to maintain coupling of reaction rates. Based on this idea and under the assumption that elementary biochemical reactions can be modeled via mass action kinetics (without neglecting the effect of enzymes), here we propose a novel means to analyze metabolic profiles based on the concept of constrained maximal correlation coefficient. We use this approach to analyze and characterize the role of metabolites in a network that captures the reaction rate coupling. First, by using a paradigmatic model of the tricarboxylic acid (TCA) cycle, we investigate the effect from departures of the assumption of mass action on the identified reaction coupling and couplings of reactant complexes. We then show that Stoichiometric Correlation Analysis (SCA) can be employed to perform cross-species comparison of the TCA cycle and amino acid synthesis pathways. In addition, we demonstrate that the proposed approach can be used to mechanistically understand the agronomicaly important process of domestication, here, in the case of wheat as well as in tomato and strawberry.

## Materials and methods

### Description of the approach with the underlying assumptions and principles

#### Maximal correlation

**Figure 1:**
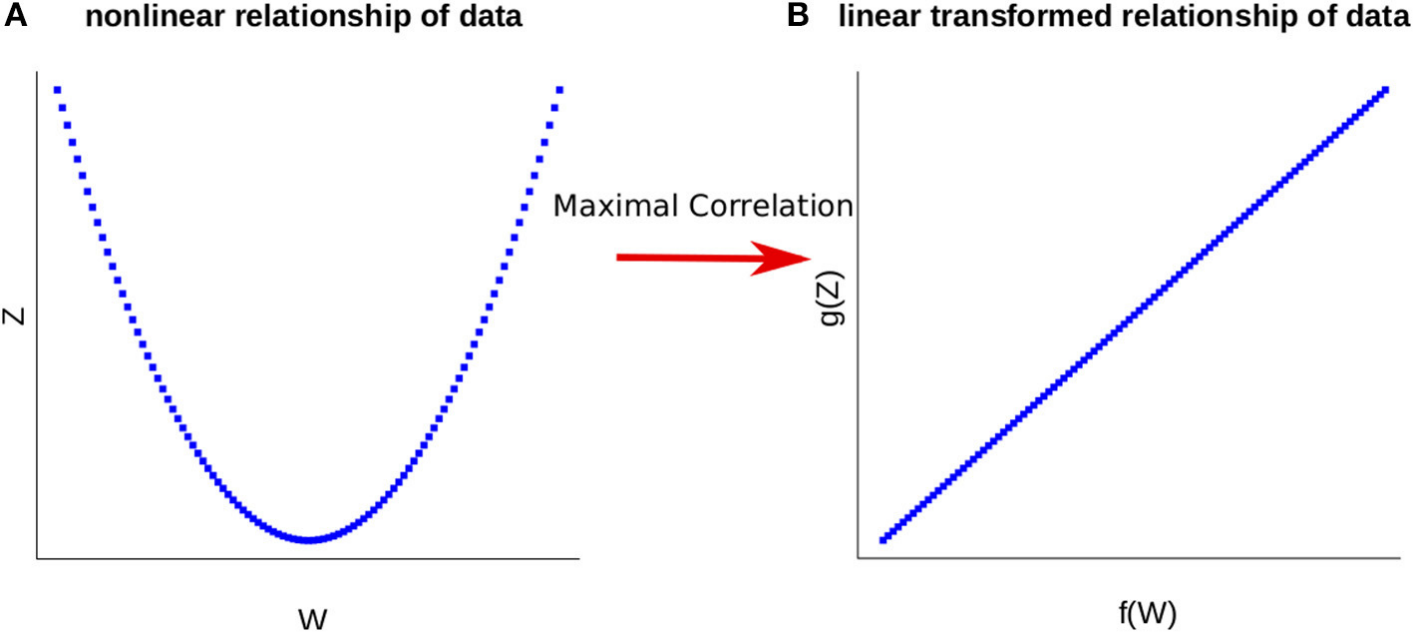
Representation of the Maximal Correlation. **(A)** The relationship of the variables W and Z is nonlinear. **(B)** Employing maximal correlation finds the functions f and g. These allow the transformation of the data and capture the underlying relationship between the variables W and Z.

Modern applications, particularly in computational biology, often consider a large number of variables involved in nonlinear (pairwise) relationships. The maximal correlation coefficient ρ, between a pair of random variables *W* and *Z*, introduced by Gebelein (1941) and already extensively studied by Lancaster (1957) and Rényi (1959), is defined as:

(1) $$\begin{array}{r} \rho =\text{sup}\left\{\frac{cov\left(f\left(W\right),g\left(Z\right)\right)}{\sqrt{V\left(f\left(W\right)\right)V\left(g\left(Z\right)\right)}}|V\left(f\left(W\right)\right)>0,V\left(g\left(Z\right)\right)>0\right\}, \end{array}$$

where the supremum is taken over all functions *f* of *W* and *g* of *Z* with finite variances, i.e., *V*(*f*(*W*)) > 0 and *V*(*g*(*Z*)) > 0. Maximal correlation then infers (non-linear) transformations of two random variables by maximizing their pairwise correlation (see Figure 1 for illustration). We note that *W* and *Z* are independent if and only if ρ = 0, relating maximal correlation to mutual information (see Introduction).

There exist efficient algorithms to compute maximal correlation for both discrete (Breiman and Friedman, 1985) and continuous (Lancaster, 1957) random variables. Direct application of these algorithms for calculation of maximal correlation to time-resolved metabolic profiles is hampered since: (1) metabolic profiles are quantitative (i.e., continuous variables), as they capture the content of metabolic pools in biological systems; therefore, any decision to move to a range of values (e.g., small, medium, large, as it is done in discretization), will lead to drastic simplification, and (2) time-resolved metabolic profiles include relatively few time points, rendering the calculation of maximal correlation based on contingency table challenging (e.g. Nguyen et al. (2014) analyzed maximal correlation with at least 100 data points which is still not available for metabolomics data).

### Stoichiometric correlation analysis and the principle of metabolic network robustness

**Figure 2:**
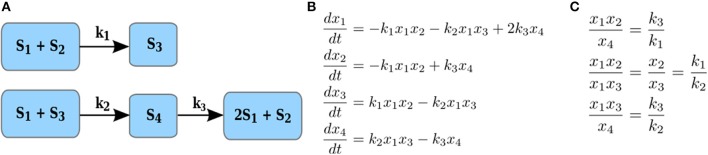
Illustration of reaction couplings. **(A)** Network with four components, S1–S4, and three reactions with rate constants k1–k3; **(B)** A system of ODEs with mass action kinetics describing the change in concentration of each of the four components. **(C)** Couplings of reaction rates by invoking the steady-state assumption for the system of ODEs in **(B)**.

Here we define a constrained version of the maximal correlation coefficient which is motivated by modeling of metabolic networks and the principles of their operation. A metabolic network is a collection of metabolites and biochemical reactions through which they are transformed and/or exchanged with the environment. For instance, the network on Figure 2A transforms four metabolites, *S*_1_to *S*_4_ via three reactions. Each reaction takes a non-negative linear combination of reactants metabolites, called substrate complex, and transforms it into a product complex, i.e., a non-negative combination of product metabolites. The coefficients in the non-negative linear combination denote the stoichiometry with which a metabolite enters a reaction as a substrate and/or product. For instance, in Figure 2A, *S*_1_ + *S*_2_ is the substrate complex of reaction *r*_1_ and 2*S*_1_ + *S*_2_ is the product complex of reaction *r*_3_. The difference between the stoichiometry of the product and substrate complexes defines a reaction vector stoichiometry gathered in the stoichiometric matrix *N*. In other words, the entry α_*ij*_ of the stoichiometric matrix *N* contains the molarity (integer number) with which metabolite *i* is involved as a substrate or product in the reaction *j* (Heinrich and Schuster, 1996).

The change in the levels of *n* metabolites, *x*_1_, …, *x*_*n*_ can then be described by an ordinary differential equation (ODE), $\frac{dx}{dt}=N*v\left(x,k,t\right)$ where *N* denotes the stoichiometric matrix with dimensions *m* × *n*, with m the number of metabolites and n the number of reactions, *v* denotes the reaction rate functions, *x*, the concentrations of the considered metabolites, *k*, the parameters on which the reaction rates depend, and *t* stands for time (Nägele et al., 2016). Even in the simple case of mass action kinetics for a network of bimolecular reactions, the reaction rates, gathered in the time-dependent vectors, *v*(*x, k, t*), are described by a non-linear function (Horn and Jackson, 1972). Metabolic reactions usually are not spontaneous and are catalyzed by enzymes. Every enzymatic reaction can in turn be divided into elementary reactions. Elementary reactions consider the formation and dissociation of enzyme-substrate complexes and provide the possibility for modeling variety of regulatory mechanisms (Segel, 1975). Elementary reactions can be effectively modeled with mass action, since they can be cast to explicitly consider the action of the enzyme (as in the derivation of the Michaelis-Menten kinetic). This was the approach taken in the large-scale model of *E. coli* (Khodayari and Maranas, 2016) and some of the subsystems in the models of photosynthesis (Arnold and Nikoloski, 2011). Therefore, due to the combined effect of multiple reactions and their regulation, metabolic data profiles can be regarded as observations from non-linear dynamics of metabolic networks.

Let *x*_*i*_ denote the concentration of a substrate component *S*_*i*_ (i.e., metabolite or enzyme). The rate of reaction *j* with a substrate complex $\underset{i}{\sum }\alpha_{ij}S_{i}$ with α_*ij*_ > 0 where α_*ij*_ is the stoichiometry with which *S*_*i*_ enters the substrate complex of reaction *j*, under mass action kinetics is then expressed as $k_{j}\prod_{i}x_{i}^{\alpha_{ij}}$, where *k*_*j*_ denotes a rate constant. For instance, the rate of reaction with rate constant *k*_1_ in Figure 2A is given by *k*_1_*x*_1_*x*_2_, since *S*_1_ and *S*_2_ enter this reaction as substrates, each with stoichiometric coefficients of one; similarly, the rate of reaction with rate constant *k*_3_ is given by *k*_3_*x*_4_, since *S*_4_ enters the reaction as a substrate with a stoichiometric coefficient of one.

To arrive at our approach termed Stoichiometric Correlation Analysis (SCA) we rely on the observation that metabolic networks, as part of inter-related cellular systems (e.g. transcription, translation, and signaling), operate toward providing robust functionality (Kitano, 2007; Wilson, 2013). We translate the robust functionality in the ability to ensure coupling of reaction rates (Millard et al., 2017). To formalize SCA, we provide the following definitions:

**Definition 1:** Two elementary reactions, *p* and *q*, have coupled rates under mass action kinetics if for any steady-state concentration of the participating components, gathered in *x*,

$$\begin{array}{l} \frac{k_{p}\prod_{i}x_{i}^{\alpha_{ip}}}{k_{q}\prod_{i}x_{i}^{\alpha_{iq}}}=\frac{k_{p}}{k_{q}}\prod_{i}x_{i}^{\alpha_{ip}-\alpha_{iq}}=\;\gamma_{pq},\text{where }\gamma_{pq}\text{is a constant}. \end{array}$$

For instance, at any steady state for the network in Figure 2A, whereby the equations in Figure 2B all equal 0, i.e., $\frac{dx_{i}}{dt}=0$, reactions *r*_1_ and *r*_2_, *r*_1_ and *r*_3_, as well as *r*_3_ and *r*_2_ have coupled rates (see Figure 2C). We note that the same definition can be extended to hold in states which are not necessarily equilibrium points, allowing the treatment of time-series data. We would like to note that the coupling of reaction rates may lead to coupling of component concentrations which are not apparent by directly inspecting the reaction networks. For instance, due to the coupling of reactions *r*_1_ and *r*_2_, the concentration of components *x*_1_ and *x*_2_ also are coupled, i.e., are proportional to each other.

Since the non-zero stoichiometric coefficients α_*ij*_ are integers in the set *I* = {1, …, 4} (Basler et al., 2012), given two disjoint sets *U*_*p*_ and *U*_*q*_ of random variables denoting the data profiles for the metabolites, we next define the stoichiometric correlation.

**Definition 2:** Given two disjoint sets of random variables *U*_*p*_ and *U*_*q*_, denoting two sets of metabolic profiles, the stoichiometric correlation is given by:

(2) $$\begin{array}{r} sup\left\{\frac{cov\left(f\left(U_{p}\right),g\left(U_{q}\right)\right)}{\sqrt{V\left(f\left(U_{p}\right)\right)V\left(g\left(U_{q}\right)\right)}}|V\left(f\left(U_{p}\right)\right)>0,V\left(g\left(U_{q}\right)\right)>0\right\}, \end{array}$$

with

$$\begin{array}{l} f\left(U_{p}\right)=\overset{\left|U_{p}\right|}{\underset{i=1}{\sum }}\beta_{ip}log\left(x_{i}\right),\beta_{ip}\in I \end{array}$$

and

$$\begin{array}{l} g\left(U_{q}\right)=\overset{\left|U_{q}\right|}{\underset{i=1}{\sum }}\eta_{iq}log\left(x_{i}\right),\eta_{iq}\in I. \end{array}$$

If *U*_*p*_ and *U*_*q*_ include the random variables corresponding to the metabolite levels in the substrate complexes of reaction *p* and *q*, respectively, the proposed definition of the stoichiometric correlation is a direct consequence of Definition 1, where the functions *f*(*U*_*p*_) and *g*(*U*_*q*_) are the logarithm of the rate of the reactions *p* and *q* under mass action kinetics, respectively. The presence of coupled rates in mass action for reactions *p* and *q*, after taking the logarithm, leads to stoichiometric correlation of value one for *U*_*p*_ and *U*_*q*_. This observation pinpoints the main principle on which SCA relies.

If there exist multiple vectors β and η, yielding the same value of the stoichiometric correlation, we consider the one of smallest magnitude ||β+η_2_||. Therefore, stoichiometric correlation can be regarded as constrained maximal correlation, where the constraints pertain to the limited set of values that the entries of β and η are allowed to take following the stoichiometry of reactants. The transformation used in the constrained maximal correlation is explicitly non-linear, since the function *f* involves logarithms.

Clearly, the reverse direction also holds and can be used to draw hypotheses about the couplings in reaction rates and substrate complexes in a given metabolic network. To this end, we focus on the statistically significant stoichiometric correlations larger than a threshold value of 0.8 (to account for effects of noise and small deviations from coupling of reaction rates, per Definition 1). Note that since *U*_*p*_ and *U*_*q*_ are disjoint sets of random variables denoting the data profiles of metabolites, the entries of β and η are positive. For instance, given several steady-state measurements for the components in network on Figure 2A, the stoichiometric correlations with *U*_*p*_ = {*S*_1_, *S*_2_} and *U*_*q*_ = {*S*_4_} is one with coefficients in β and η equal to one. Similar conclusions can be drawn for all components involved in the coupled reaction rates given in Figure 2C. The two definitions provide the basis for SCA: Since majority of reactions in real-world metabolic networks are mono- or bi-molecular (i.e., include one or two substrates), we determine the stoichiometric correlation, per Definition 2, between any two disjoint subsets of random variables of cardinality at most two. The implementation can either be achieved by: (1) solving a non-linear program with constraints for the coefficients β, η ∈ *I* or (2) generating all subsets of at most two variables with different contribution due to stoichiometry, and determining the Pearson correlation coefficient only between the disjoint subsets. Since the number of available metabolic profiles from time-resolved studies usually does not exceed 100, the second alternative can be efficiently implemented with appropriate parallelization (see the code Schwahn et al., 2017). The significance of the stoichiometric correlation can be readily estimated by permutation tests after adjusting for multiple hypothesis testing.

### Implementation of SCA

Given a data set of *n* metabolites over *c* samples (i.e., each representing a particular time point in an environment), we implemented SCA by determining: (1) the Pearson correlation *r*(log(x_*i*_), log(*x*_*j*_)), for all couples 1 ≤ *i* ≠ *j* ≤ *n* of metabolic profiles, (2) the values for *a, b* ∈ {1, 2, 3, 4} that maximize the Pearson correlation between *a* log(*x*_*i*_) + *b* log(*x*_*j*_) and *x*_*k*_ for every triple of metabolic profiles, (3) the values for *a, b, c, d* ∈ {1, 2, 3, 4} that maximize the Pearson correlation between *a* log(*x*_*i*_)+*b* log(*x*_*j*_) and *c* log(*x*_*k*_)+*d* log(*x*_*l*_) for every quadruple of metabolic profiles. In addition, we determined the statistical significance for each of the maximum correlations. We used the R package Hmisc (Harrel and, 2015) to calculate the correlation and associated *P*-values. In addition, we adjusted the *P*-values using Benjamini-Hochberg multiple hypotheses testing correction. We considered stoichiometric correlations with adjusted *p*-values below α = 0.05 as significant.

The code and one example can be found on GitHub: https://github.com/KSchwahn/Stoichiometric-correlation (Schwahn et al., 2017).

### Models

Metabolite levels were simulated with three different models using Michaelis-Menten kinetics (Singh and Ghosh, 2006), mass action kinetics and extended mass action kinetics with metabolite-enzyme complexes (Khodayari et al., 2014). The Michaelis-Menten based model contains 11 reactions and 12 metabolites and simulates the metabolite levels within the TCA cycle of *E. coli* growing on glucose. The synthetic reaction (SYN) and the biomass metabolite (biosyn) were removed, as a comparable reaction and metabolite were not present in the other two analyzed models. The modified Michaelis-Menten model contains therefore 11 metabolites and 10 reactions. All kinetic parameters remained unchanged. The mass action based models contain the TCA cycle of the *E. coli* model of Khodayari et al. (2014). The solely mass action based model contains 23 metabolites and 22 reactions after splitting each reaction into a forward and backward reaction. The second model includes the simulation of metabolite-enzyme complexes based on mass action kinetics. The model contains 114 irreversible reactions and a total of 80 metabolites, enzymes and metabolite-enzyme complexes.

The change of concentration was simulated with each model over a time course of 1,280 min. The initial concentration of the metabolites, metabolite-enzyme complexes and enzymes was randomly assigned for each of the 10 repetitions from the range of the minimum and maximum metabolite concentration reported in Khodayari et al. (2014). The same set of 11 metabolites, present in each model, was then used for the calculation of stoichiometric correlations with the SCA approach. All simulations were performed in MATLAB 2015a (MATLAB, 2015).

### Metabolic data profiles

We applied SCA to several publicly available metabolomics data sets, including metabolic profiles from *Arabidopsis thaliana* obtained from Caldana et al. (2011) and *Escherichia coli* from Jozefczuk et al. (2010). The first consists of data profiles of 92 metabolites over eight conditions measured over 22 time points with 6 replicates each (light and dark at 4, 21, and 32°C, as well as low light at 21°C and high light at 21°C; high light was discarded as it contains less time points), while the second includes 196 metabolites over five conditions measured over 12 time points with three biological replicates per time point and three technical replicates each (cold stress, heat stress, oxidative stress, lactose and control condition).

We also used the metabolomics data from a recent evolutionary metabolomics study (Beleggia et al., 2016). The study identified and quantified 51 metabolites from nine compound classes in the three taxa of wheat, namely, wild emmer, emmer, and durum wheat. The metabolites were measured in kernels of 12 accessions from wild emmer, 10 from emmer, and 15 accessions from durum wheat, whereby the measurements contain three biological replicates with three technical replicates each. Like the other data sets used here, the metabolic profiles in the wheat taxa were assessed by gas chromatography mass spectrometry. To allow comparability between taxa, we used only the 22 metabolites, from four compound classes, which were detected across all accessions.

Moreover, we included metabolomics data from six different tomato species, namely *S. chmielewskii, S. habrochaites, S. lycopersicum, S. pimpinellifolium, S. neorickii*, and *S. pennellii* (Schauer et al., 2005). We considered data from the ripe fruit in this study. These data were included to further test our assumption about the effect of domestication on reaction coupling. Altogether, we compared 43 metabolites form the tomato data. The *S. lycopersicum* metabolomics measurements were obtained from the study of Schauer et al. (2006) and contain 108 replicates from the year 2001 and 84 replicates from the year 2003, whereas the remaining data were obtained from Schauer et al. (2005) and contain six replicates for each of the five species.

To have a more comprehensive comparative analysis pertaining to domestication, we included further data of wild strawberry accessions (*F. vesca*) and a domesticated strawberry species (*F. ananassa*) (Ulrich and Olbricht, 2013). Overall, 19 different metabolites had complete measurements to be included in the analysis. The data set contains measurements from 32 samples of *F. vesca* and 10 samples of *F. ananassa*. This data set in comparison to the other data sets contains specifically the volatile organic compounds extracted from the strawberry fruits.

## Results and discussion

### Stoichiometric correlation analysis with a paradigmatic model of the TCA cycle

From the derivation of our SCA, it follows that the findings based on the constrained correlation of metabolic data profiles reflect the apparent couplings of elementary reaction rates, assumed to obey mass action kinetic. In addition, the findings reflect the additional couplings which cannot be directly related to reaction rates but are direct consequence of them (e.g., components *S*_1_ and *S*_2_ in the network on Figure 2A are coupled due to the coupling of the rates of reactions *r*_1_ and *r*_2_). We note that every enzymatic reaction ∑_*i*_ α_*ij*_*S_i_* → ∑_*i*_α′_*ij*_*S_i_* (α_*ij*_/α′_*ij*_ are the stoichiometry with which *S*_*i*_ enters the substrate/product complex of reaction *j*, respectively) can be rewritten to include the action of an enzyme ∑_*i*_ α_*ij*_*S_i_* + *E* ⇄ *SE* ∑_*i*_α′_*ij*_*S_i_* + *E* (E denotes the enzyme and SE the substrate-enzyme complex), so that the elementary reaction can be still modeled with mass action kinetic. Therefore, SCA can also include the effect of enzyme action. However, while this approach provides a way to model Michaelis-Menten kinetic which accounts for enzyme saturation, it does not explicitly consider the Michaelis-Menten form for the reaction kinetic.

To investigate the effects of the departure from the mass action kinetic for the considered reactions (with and without accounting for enzyme action), we considered three models of the tricarboxylic acid (TCA) cycle. All three models include the same metabolites, and differ only with respect to whether or not they include the effect of enzyme action and if they use mass action kinetic or the more involved functional forms of the Michaelis-Menthen kinetic. All reactions are considered reversible, and they are split into irreversible reactions in the cases in which mass action kinetic was employed. We used the TCA cycle model embedded in the kinetic model of *E. coli* (Khodayari et al., 2014). There are two parameterized variants for this model, one that includes mass action kinetic without enzyme action, and the other one which explicitly considers the formation of substrate-enzyme complexes. In addition we used a model of the TCA cycle with reversible Michaelis-Menten kinetic of the reaction rates (Singh and Ghosh, 2006).

**Figure 3:**
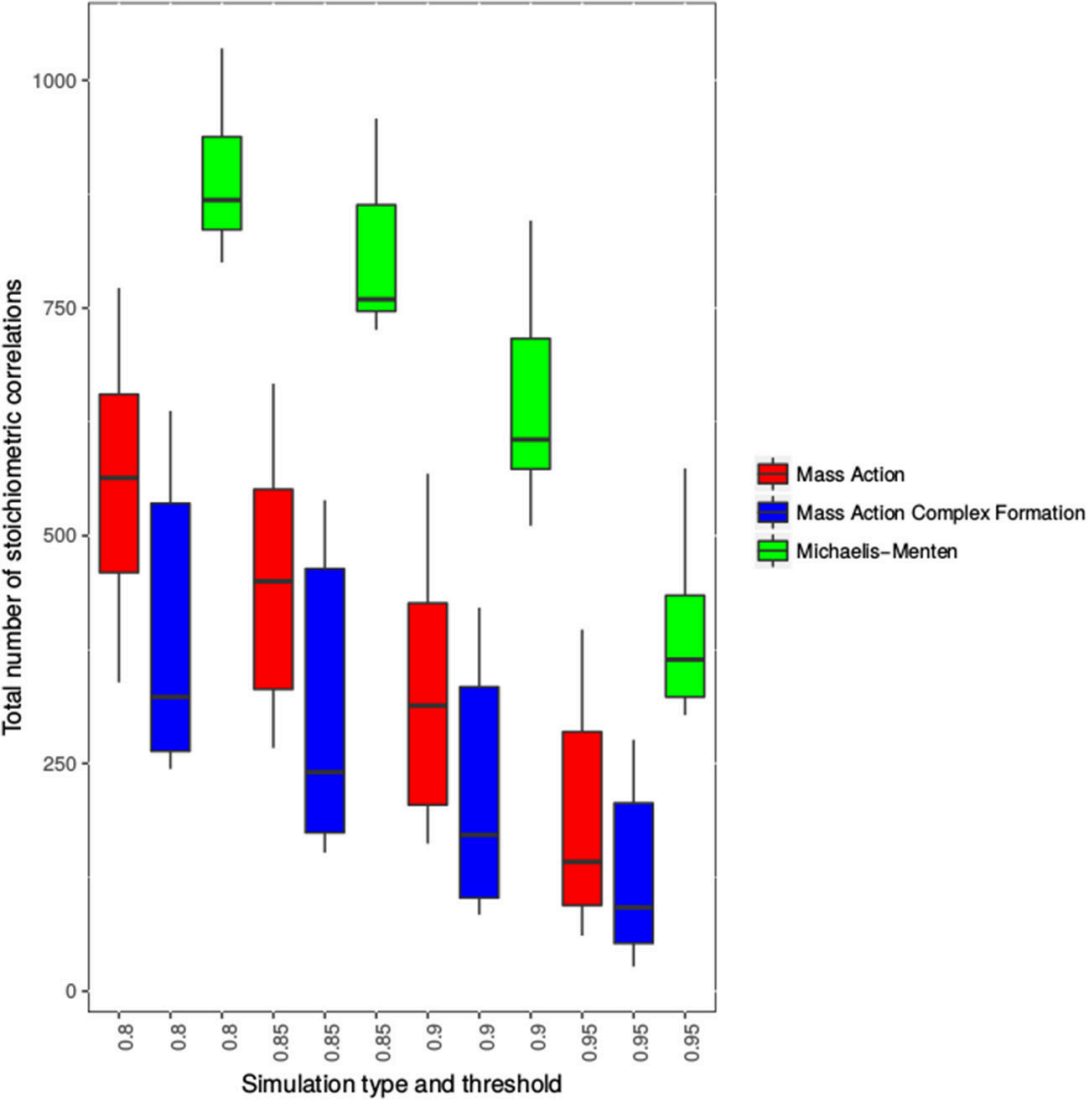
Distribution of the number of stoichiometric correlations for three models of the TCA cycle. Shown are the distributions of the total number of stoichiometric correlations at four thresholds 0.8, 0.85, 0.9, and 0.95. The distributions for the mass action simulation are shown in red, the distributions for the substrate-enzyme complex mass action simulation are shown in blue, whereas the Michaelis-Menten simulation of the TCA cycle is shown in green.

To conduct the comparative analysis, we simulated the models metabolite concentrations with physiologically relevant randomly chosen initial values. The simulation time ranged from 0 to 1,280 min and metabolite concentrations were obtained at 21 time points identical to those used in the study of Caldana et al. (2011) (which we employ later in the empirical analysis). The simulated metabolite concentrations were used to calculate the stoichiometric correlations for 11 metabolites for each simulation and model separately. The distribution of the total number of stoichiometric correlations over 10 repetitions of the procedure is shown in Figure 3, and all stoichiometric correlations (pairs, triplets and quadruples) are provided in Supplemental Table S1.

**Figure 4:**
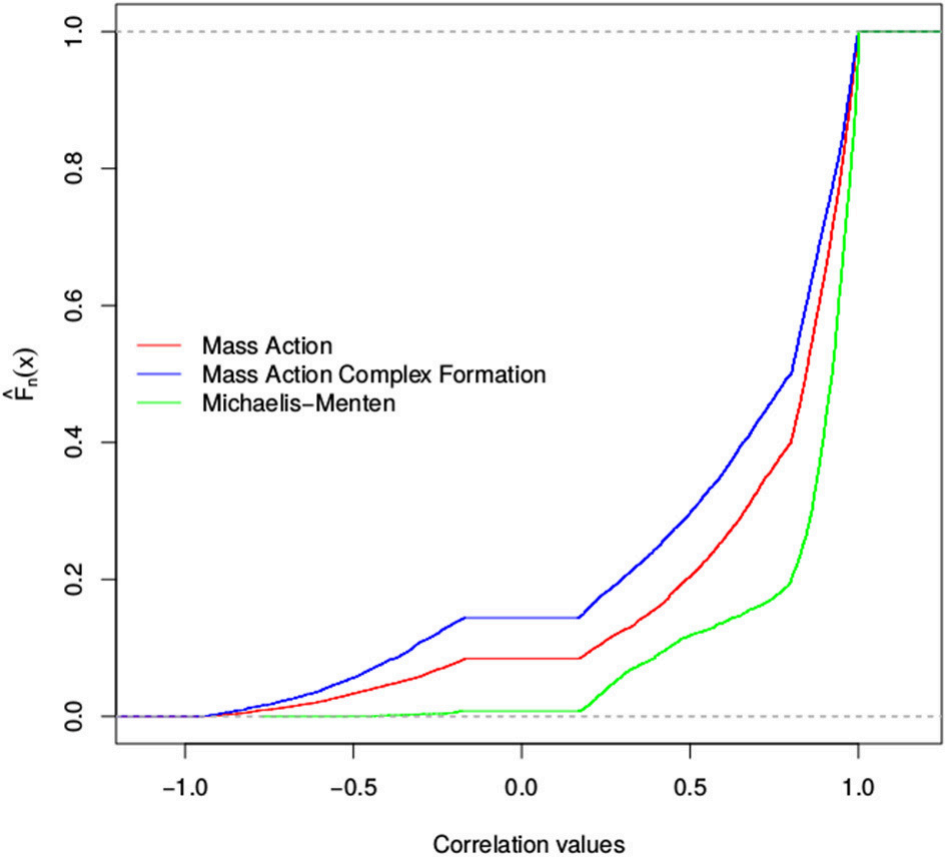
Empirical cumulative distribution function of stoichiometric correlations for three models of the TCA cycle. Shown is the Empirical cumulative distribution of the total number of stoichiometric correlations of simulations of the TCA cycle. The distribution for the mass action simulation is shown in red, the distribution for the substrate-enzyme complex mass action simulation is shown in blue, whereas the Michaelis-Menten simulation is shown in green.

We found that the total number of stoichiometric correlations between the models with mass action kinetic was more similar with the increase in the considered threshold. In fact, at a threshold of 0.95, the distributions of the total number of stoichiometric correlations between the mass action models with and without the consideration of enzyme action largely overlapped. However, the consideration of reversible Michaelis-Menten kinetic results, on average, in at least three-fold increase in the total number of stoichiometric correlations (see Figure 3). These findings were supported by the results of the empirical cumulative distribution function (see Figure 4). The distribution of the Michaelis-Menten simulations are shifted to the right and show a higher proportion of correlations above 0.8. In addition, we report the quintiles of the correlation values in Supplemental Table S2.

Therefore, in the case of the TCA cycle models, we concluded that the findings from the assumption that the network is composed of elementary reactions modeled with mass action do not differ upon consideration of enzyme action. In these cases, the couplings corresponding to the stoichiometric correlations reflect the underlying reaction couplings. In contrast, the usage of Michaelis-Menten kinetic results in a considerably larger number of stoichiometric correlations, which cannot be brought in direct correspondence to the coupling of reaction rates and are challenging to mechanistically explain.

### SCA demonstrates differences in the stringent response between *E. coli* and *A. thaliana*

The stringent response is one of the most important regulatory systems used by bacteria to adapt to environmental stresses. Upon sensing the environmental change, like nutrient limitation, the organism starts a series of reactions to redirect its metabolic fluxes. The stringent response is mediated by guanosine 3′,5′-bis(pyrophosphate) (ppGpp) whose level is controlled by two enzymes, RelA and SpoT (Traxler et al., 2008). ppGpp has overall a large influence on several metabolic pathways and transcription and translation (Gallant, 1979; Mizusawa et al., 2008). The effect in metabolism involves the pathways of nucleotides, glycolytic intermediates, carbohydrotes, lipids and fatty acid synthesis. It has been reported that there is evidence that the stringent response is evolutionary conserved from bacterial to photosynthetic bacterial to higher plants (Sugliani et al., 2016). Four homologs of these RelA and SpotT have been identified in Arabidopsis, and their role in green tissues and flower development has been well characterized (Masuda et al., 2008; Mizusawa et al., 2008). Since all these plant proteins are targeted to the chloroplast, it has been suggested that they control the stringent response in photosynthesizing organisms through mechanisms that may mimick those in bacteria. However, it remains unclear to what extent the molecular role of the homologs in *A. thaliana* are equivalent to those in *E. coli*.

To help answer this question, we analyzed the set of metabolites from the TCA cycle and the amino acid synthesis pathways from the two model organisms. We used these metabolites since ppGpp controls transcription and translation, which is ultimately reflected in the levels of amino acids. Moreover, a comparative analysis between the two organisms is only meaningful for the same set of metabolites. Altogether, we used the publicly available data profiles of three metabolites from the TCA cycle (i.e., malate, succinate, and fumarate) as well as 16 amino acid measured over seven and five conditions in *A. thaliana* and *E. coli* (see Materials and Methods).

The degree of coupling for metabolite *S* can be defined as the number of stoichiometric correlations above a given threshold τ in which the metabolite *S* participated. Based on the derivation of SCA, a higher degree of coupling on the same set of metabolites then implies maintenance of more coupled reaction rates over a set of studied conditions in one organism in comparison to another. We considered the significant stoichiometric correlations (*p* ≤ 0.05, Benjamini-Hochberg corrected) 0.8, 0.85, 0.9, and 0.95, and compared them to classical Pearson correlations (Table 1). The quintiles of correlation values were additionally reported in Supplemental Table S2.

**Table 1:** Overview of the number of significant stoichiometric correlations at the considered thresholds for metabolic profiles of the stringent response in *E. coli* and *A. thaliana*.

		**Stoichiometric Correlation**				**Pearson Correlation**
**Threshold**	**Organism**	**Total**	**Pairs**	**Triplets**	**Quadruples**	**Pairs**
0.80	*A. thaliana*	3,419	13	579	2,827	24
	*E. coli*	3,301	9	517	2,775	10
0.85	*A. thaliana*	2,500	8	398	2,094	18
	*E. coli*	1,921	6	285	1,630	7
0.90	*A. thaliana*	1,821	6	285	1,530	15
	*E. coli*	597	1	76	520	2
0.95	*A. thaliana*	1,137	6	188	943	7
	*E. coli*	2	0	0	2	0

*The total number of stoichiometric correlations is divided into three groups based on whether they involve pairs, triples, or quadruples of metabolites. Additionally, the number of significant Pearson correlations found in the dataset is shown*.

For the purpose of comparison, at all threshold values and in both species, we observed a decrease on the number of significant stoichiometric correlations for pairs of metabolites, compared to Pearson correlation (i.e.,|*U*_*p*_| = |*U*_*q*_| = 1). The reduction in the number of significant stoichiometric correlations for metabolite pairs can be explained by the monotonic transformation of metabolite profiles. We would like to emphasize that the result does not suggest that metabolites are linearly related, which would be contrast to what is expected from mechanistic understanding of metabolism.

However, SCA allows the analysis of stoichiometric correlations due to triples and quadruples of metabolites, which provides information about the presence of non-linear relationships via the couplings of reaction rates. For all considered thresholds, applying SCA with the *E. coli* data set resulted in a smaller number of stoichiometric correlations on triples and quadruples than the data set of *A. thaliana* (Table 1). For instance, at a threshold of τ = 0.85, *E. coli* yielded 285 significant stoichiometric correlations due to triples while *A. thaliana* resulted in 398 such correlations; similarly, *A. thaliana* contained three-fold the number of stoichiometric correlations resulting from quadruple at τ = 0.9 in comparison to *E. coli*. Therefore, based on these results we concluded that there was a stronger coupling of reaction rates of *A. thaliana* in comparison to *E. coli* during the stringent response.

Additionally, we can investigate overlapping pairs, triple and quadruple for each threshold. The small similarity of SCA findings was reflected in 65 and 442 stoichiometric correlations due to triple and quadruple, respectively, shared between the two species at a threshold value of 0.8 (see Supplemental Table S3). In line with this observation, the participation of metabolites in the stoichiometric correlations largely differed between the two species, as manifested in the lack of association between the metabolite coupling degrees. For instance, at a threshold value of 0.85, the metabolites with the largest coupling degrees in *E. coli* were: phenylanine, threonine, proline and lysine, while in *A. thaliana* they included: isoleucine, leucine, tyrosine and lysine (see Supplemental Table S4). It must be noted that these results and interpretations warrant caution since the metabolite profiles from *A. thaliana* were obtained from entire Arabidopsis rosette rather than from isolated chloroplast, which may bias the drawn conclusions. The analysis can be conducted with compartment-specific metabolic profiles once they become available.

### SCA shows that domestication in wheat is associated with loss of regulatory couplings

Domestication of tetraploid wheats, *Triticum turgidum L*., is an important evolutionary event for the human development. Emmer (*T. turgidum ssp. dicoccum*) was domesticated from wild emmer (*T. turgidum ssp. dicoccoides*) around 12,000 years ago (Nesbit and Samuel, 1998). Free-threshing tetraploid wheats (*T. turgidum ssp. turgidum*) subsequently originated from emmer, followed by the selection of durum wheat (*T. turgidum ssp. turgidum convar. durum*). Therefore, it has been suggested that the evolution of tetraploid wheats consists of at least two steps: primary domestication, from wild emmer to emmer, and secondary domestication, from emmer to durum wheat (Gioia et al., 2015).

Since important domestication-associated traits (e.g., the increase in seed size, the loss of dormancy Gepts and Papa, 2002) often necessitate alteration of metabolic process, we asked if application of SCA to metabolic profiles can be used to quantify the effect of domestication with respect to loss or gain of regulatory couplings. To this end, we used recently analyzed data about the phenotypic variation of primary metabolites in the kernels from three *T. turgidum* populations that represent both the primary and secondary domestication (Beleggia et al., 2016). Beleggia et al. (2016) determined that there were changes in content of specific metabolites, particularly amino acids and unsaturated fatty acids, associated with the primary and secondary domestication events. The resulting metabolic profiles of accessions within each taxon were also employed to construct Pearson correlation networks. Based on various properties of the correlation networks (e.g., shared correlations, centrality of metabolites) it was concluded that the difference between wild emmer and emmer was larger than the difference between wild emmer and durum wheat. In addition, it was found that durum wheat contained a larger number of significant correlations, followed by wild emmer and emmer. Therefore, surprisingly, the results from Pearson correlation analysis captured contrasting findings in comparison to the evolutionary distance between the three analyzed taxa.

**Figure 5:**
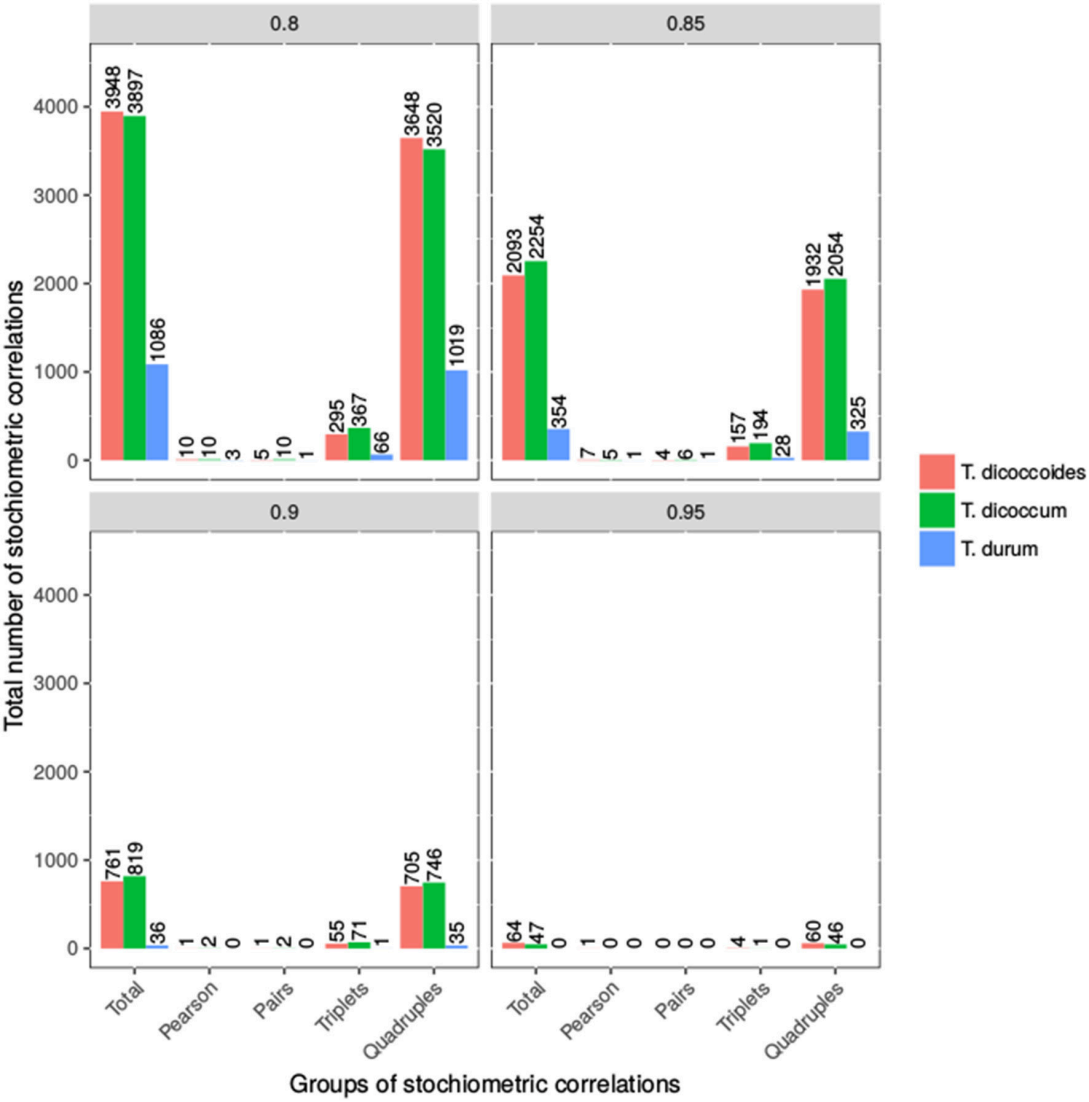
Number of Stoichiometric Correlations of Wheat taxa. Shown is the number of stoichiometric correlations at the four thresholds 0.8, 0.85, 0.9, and 0.95. The bars represent the total number of stoichiometric correlations, pairs, triplets and quadruples for all three wheat taxa. The exact values are shown above the bars.

We applied SCA to contents of 22 metabolites from four compound classes (i.e., amino acids, sugars, organic acids, and alcohols) within each population at four threshold values (see Materials and Methods). These metabolites were selected based on their presence in every of the analyzed accessions to allow comparative analysis of the populations without the need of imputation as well as assumptions about the reasons for absence of detected metabolite. The number of stoichiometric correlations due to triples shared between emmer and wild emmer was the highest, followed by that between durum and wild emmer (at threshold of 0.8). At a threshold value of 0.85, both emmer and wild emmer had one overlapping triple with durum. However, at a threshold value of 0.8, durum wheat shared more stoichiometric correlations due to quadruples with wild emmer than emmer. At thresholds of 0.85 and 0.9, durum shared the same number of quadruples with emmer and wild emmer. In all cases, only stoichiometric correlations due to quadruples were shared between all three populations (e.g., stoichiometric correlations at threshold of 0.85, Supplemental Table S6). Overall, we observed more triples and quadruples in *T. dicoccum* and *T. dicoccoides* in comparison to *T. durum* (see Figure 5). The observation was supported by the quintiles of the correlation values shown in Supplemental Table S2.

This finding implied that the loss of traits due to domestication and increase in seed size were associated with an overall loss of reaction couplings reflected in the smaller number of stoichiometric correlations in durum wheat in comparison to (wild) emmer (Supplemental Tables S5–S7). The metabolites involved in the largest number of stoichiometric correlations above a threshold value of 0.85 in wild emmer included glycine, threonine, aspartate, serine and glutamate; in emmer, these metabolites included serine, leucine, threonine, and glutamate, while in durum wheat they consisted of fructose, glucose, glutamate, and asparagine (see Supplemental Table S7). Altogether, the application of SCA identified a shift in importance of regulatory role of sugars in comparison of organic and amino acids which is in line with the increase in seed size due to the need for more cell wall components.

To further validate our results from the three wheat taxa, we included data of six different tomato species into the analysis. We compared the domesticated *S. lycopersicum* (M82) to the group of the other five species, as their fruits drastically differ from those of M82 (Schauer et al., 2005). However, it has to be noted that there is no clear linage from the undomesticated plants to the M82. Additionally, the combination of the different tomato species might result in an inclusion of additional noise. Nevertheless, it is a necessary step to have the needed amount of replicates per metabolite. Overall, the tomato data set contains 43 metabolites common to the analyzed species. In line with the results of wheat, we observed fewer stoichiometric correlations for M82 than for the undomesticated wildtype tomato (Table 2, Supplemental Table S8). The exception is the threshold of 0.8; in this case, the M82 has roughly 4,000 more pairs, triples and quadruples than the wild type tomato. At a threshold value of 0.85 the M82 has still around 300 stoichiometric correlations more than the wild type species. With increasing threshold, however, the number of significant stoichiometric correlations decreases in M82 more than in the wild type. This finding was reflected in the different quintiles of the correlation values for the two species (Supplemental Table S2). The metabolites with the largest number of stoichiometric correlations above a value of 0.9 in wildtype tomato are erythritol, cysteine, succinic acid and beta-alanine, while in M82, they include: leucine, putrescine, dehydroascorbic and sucrose (Supplemental Table S4).

**Table 2:** Overview of number of significant stoichiometric correlations at different thresholds for the considered tomato and strawberry species.

		**Stoichiometric Correlation**				**Pearson Correlation**
**Threshold**	**Organism**	**Total**	**Pairs**	**Triplets**	**Quadruples**	**Pairs**
0.80	Tomato wildtype	19,519	8	1,245	18,266	27
	M82	23,571	15	1,824	21,732	12
	*F. vesca*	1,346	6	204	1,136	6
	*F. ananassa*	2,374	12	433	1,929	5
0.85	Tomato wildtype	9,291	5	588	8,698	20
	M82	9,539	5	688	8,846	4
	*F. vesca*	504	2	73	429	3
	*F.ananassa*	2,075	10	366	1,699	5
0.90	Tomato wildtype	3,741	3	255	3,483	11
	M82	1,493	1	112	1,380	0
	*F. vesca*	135	1	22	112	1
	*F. ananassa*	1,153	2	185	966	1
0.95	Tomato wildtype	818	1	76	741	3
	M82	21	0	0	21	0
	*F. vesca*	1	0	0	1	0
	*F. ananassa*	423	1	56	366	1

*The total number of stoichiometric correlations is divided into three groups based on whether they involve pairs, triples, or quadruples of metabolites. Additionally, the number of significant Pearson correlations found in the dataset is shown*.

A very similar scenario was considered with the strawberry accessions *F. vesca* (wild) and *F. ananassa* (domesticated and commercially available) without direct domestication lineage between the two species. In contrast to our observations in wheat and tomato, the domesticated strawberry exhibits a higher number of stoichiometric correlations above all thresholds (Table 2, Supplemental Table S9). The reason for this finding may lay in the different ploidy of the investigated organisms, namely, *F. ananassa* is an octaploid organism, whereas *F. vesca* is diploid with a rather small genome.

The application of SCA to metabolomics data from domestication implies a new principle which underlies this agronomically and evolutionary important process; namely, optimizing a given trait could be accomplished by breaking the existing regulatory mechanisms, reflected in the coupling of the biochemical reaction rates, which in turn provides a greater space of possibilities on which selection can operate.

## Conclusion

Here we proposed a constrained extension to the concept of maximal correlation, based on the concept of reaction rate coupling in networks of metabolic reactions. The concept of reaction couplings forms the core of the stoichiometric correlation analysis. The constraints in the maximal correlation are due to the values which the linear combinations of log-transformed metabolic profiles are allowed to take. SCA facilitates the comparison of data sets on the same metabolites between two scenarios with the idea of comparing and contrasting the degree of coupling. By determining the stoichiometric correlations of metabolic profiles from the TCA cycle and amino acid synthesis, we showed that *E. coli* stringent response is differently (and less strongly) controlled than that of *A. thaliana*. Therefore, while the enzymes underlying the stringent response are preserved in these two model organisms, their integration in the metabolic networks may have evolved different regulatory action. In addition, SCA can be used to investigate the differences between undomesticated and domesticated species, and to determine if the difference can be ascribed to alterations in metabolic couplings brought about by various regulatory mechanisms. Based on this idea, we demonstrate that stoichiometric correlations from metabolic profiles from natural variation in wild and domesticated species indicate that domestication is associated with loss of regulatory control. Therefore, our findings provide the basis for future flux-oriented studies toward mechanistic understanding of this important evolutionary process.

## Author contributions

ZN conceived the project and wrote the article with contribution of all authors. KS performed most of the analysis and analyzed the data. RB provided data and helped interpret the findings related to domestication. NO designed parts of the algorithm and helped with its implementation.

### Conflict of interest statement

The authors declare that the research was conducted in the absence of any commercial or financial relationships that could be construed as a potential conflict of interest. The reviewer WZ and handling Editor declared their shared affiliation.
